# A History of Battey’s Operation

**Published:** 1887-01

**Authors:** Robert Battey

**Affiliations:** Rome, Georgia


THE ATLANTA MEDICAL AND SURGICAL JOURNAL.

VOL. III. JANUARY, 1887. NO. 11.

### Original Communications.

### A HISTORY OF BATTEY'S OPERATION.

A REVIEW OF THE EARLY HISTORY OF NORMAL OVARIOTOMY—THE DIFFICULTIES ENCOUNTERED—PROFESSIONAL BRETHREN HOLDING NIGHTLY CAUCUSES AWAITING THE DEATH OF THE FIRST PATIENT IN ORDER TO ARREST THE OPERATOR—THEIR DISAPPOINTMENT—ADVICE FROM FRIENDS—OPPOSITION FROM MEDICAL JOURNALS—HIS FIRST RECOGNITION BY THE PROFESSION—IT COMES FROM THE LITERARY HUB—HOPE DAWNING—THE PRESENT ASPECTS OF THE OPERATION—ITS FUTURE—ADVICE TO YOUNG DOCTORS, ETC.

AN ADDRESS DELIVERED BEFORE THE ATLANTA SOCIETY OF MEDICINE, DECEMBER 3, 1886, BY ROBERT BATTEY, M. D., ROME, GEORGIA.

Reported Stenographically for THE ATLANTA MEDICAL AND SURGICAL JOURNAL
By W. Kay Tewksbury.

*Gentlemen of the Atlanta Society of Medicine:*

Scarcely fourteen years have elapsed since my duty called me to appear in the city of Atlanta before a medical court of my peers, the Medical Association of Georgia. The occasion of that appearance was that I felt impressed with a sense of duty to defend myself for having forsaken the well-beaten road of medical, science and wandered into a new pathway carved out by myself and thereby having given offense to the sense of propriety of my professional brethren and the offense called eminently for a clear and exhaustive statement of the reasons that compelled me to the course pursued.

On that occasion, after having spoken two hours in laying my defence before the Association, a committee was appointed to consider and report upon it. I hold in my hand a copy of the report of that committee, made by its distinguished chairman, the late Dr. Bozeman, of Atlanta. And it may not be amiss for me to read that report, as I design to call your attention first to a brief review of the early history of this proceeding and then to consider briefly its present status and endeavor to draw, as far as I may be able to do for your consideration, a horoscope of its future.

I quote from the Transactions of the Medical Association of Georgia for 1873. The following report was submitted by Dr. Bozeman:

"The committee to whom was referred the interesting paper by Dr. Battey, on the subject of the removal of the ovaries for the causes therein set forth, have not, by reason of limited time afforded to them, been able to give this important subject the thorough and diffusive investigation and discussion to which it is justly entitled. They concur, however, in according to the idea, and its successful execution by its author, the merits of originality, skill and utility. Whether it can be accepted as widely applicable to the relief of suffering from these causes must yet be confided to the cautious and judicious observation and experiment of the skillful practitioner, and they would commend the subject to the critical interest of the profession, and especially to its brilliant author, for additional experiment, and solicit reports from members of this Association on the subject at their next annual meeting.

W. F. WESTMORELAND,
C. B. NOTTINGHAM,
J. F. BOZEMAN."

The report is signed by W. F. Westmoreland, C. B. Nottingham and J. F. Bozeman. Two of these gentlemen are now dead. The report will give you, gentlemen, hardly a fair statement of the opinions and feeling of the profession of Georgia with reference to this operation at that time. I think I may say that it was hardly a fair statement. In the first place, let me say that the committee was appointed by my personal friend and co-laborer, who chanced to be President of the Association that year. Therefore there was partiality, natural partiality on my side. In the second place, two of the three members of that committee were my warm personal friends. I feel fully authorized, therefore, to say that the report was not, strictly speaking, an impartial one and did not fully represent the feeling of the profession of Georgia at that time. I will endeavor, as far as I can, to give you succinctly a statement of about what I consider a fair reflection of the state of professional feeling in Georgia at the time.

I need not say to you, gentlemen, that the offense of which I stood self-charged was in one sense a very grave one. It struck at the very foundation-stone of man's natural instinct. It was at war with man's natural sense of propriety. From our standpoint at that time, it was unsexing womankind fully and completely, a very grave offense. To give you some idea of what the state of feeling was at that time, my friend, Dr. Nottingham (the late Dr. Nottingham, of Macon), a very high-toned gentleman, as well as a most skillful and excellent physician—he was my warm personal friend—when the account of my case was first published in THE ATLANTA MEDICAL AND SURGICAL JOURNAL, he made haste to write to me. He said: "My friend, what in the world do you mean? Ovariotomy for amenorrhœa! The very proposition startles me."

Another gentleman, a leading practitioner of Southwest Georgia, about that time sent me a message by a friend. Rather a quaint character was that gentleman. He said to a friend: "When you see Battey, tell him, God bless him, I love him; but I do despise this devilish way he has of spaying women. Tell him to quit it! tell him to quit it!" These were the expressions of my friends at that time, gentlemen.

A practitioner in my own town, who was very active in his opposition to me in connection with this operation, when remonstrated with by a mutual friend, and the question argued for him, had not patience to listen to it. He said to this friend: "Doctor, do you take me for a fool? Do you suppose I would help Battey to a club to beat my brains out with?" and he turned on his heel and left him. More than that, gentlemen. Whilst engaged in nursing assiduously, as I did my first patient, spending ten days at her bedside, without leaving the house for a moment, even for a change of linen, during this time of great suspense and anxiety, in the office of one of my brother practitioners were held nightly meetings of the profession of my town, receiving reports of the condition of my patient, awaiting her demise with anxious longings in order to institute proceedings in our court and put me before the bar as a criminal. Such in a few words was the state of feeling at that time.

Of the sentiment abroad, I received in advance of the operation from the late Prof. Paul F. Eve, of Nashville, a letter expostulating with me earnestly against the proceeding, conveying a message from the Professor of Obstetrics in the University of Nashville: "Say to Battey, for God sake go slow." *Gaillard's Medical Journal*, then the *Richmond and Louisville Medical Journal*, published at Louisville, Ky., was very bitter in its denunciations of me at the time of the operation. One of the leading journals of Philadelphia cautioned me to look well to my points and be careful, and drew to my recollection the fact that an eminent Parisian surgeon had been assassinated in his carriage as he drove along the streets of Paris for a much less offense.

In the midst of this I was not without encouragement. An eminent jurist of Georgia, a citizen of Atlanta, whom Georgians delight to honor, chanced to be in New York about that time, taking council of the late Dr. Marion Sims, with reference to the health of his wife. Dr. Sims at that time was a stranger to me. I had never met him personally. He was so impressed with the statements of Dr. Sims that he sat down immediately on returning to his hotel and wrote me a letter. Dr. Sims had stated to him that he was astounded at the audacity of this operation. He said to him: "Judge, I have searched diligently the annals of medicine, and when I consider that Battey, in a small country town in Georgia, selected out a lady from the best society of his town and extirpated her ovaries, unsupported by any precedent or by any authoritative medical judgment, I find nothing in the whole history of medicine that compares with it in moral heroism." I wrote and laid my plans before Dr. Washington Atlee, then the leader of ovariotomy in the United States. He was so impressed with the magnitude of the proposition and the responsibility that attached to it, he very prudently declined to answer my letter. The first substantial recognition that I received besides that of Dr. Sims' came from the "literary hub," most unexpectedly to me. It has been, gentlemen, an enigma to me from that day to this that I should have more standing, more professional character in the city of Boston than anywhere else upon the inhabitable globe. Why this is I do not know. The Boston Gynecological Society was the first that gave recognition to me and my operation, and several of its members gave me all the aid and assistance in their power. Several other eminent physicians of Boston were equally earnest in their support and encouragement. The first recognition of my operation in print was given me in his work upon the diseases of women by Prof. Gaillard Thomas, of New York. He came out boldly in his work, espoused the operation and predicted for it a brilliant future. The publication of that edition of Dr. Thomas' work did me great good and advanced very much the acceptance by the profession the world over of this operation. It would be invidious in me, gentlemen, to mention names among my professional friends at home; most of them are still living; several of them reside in the city of Atlanta, and I feel myself under great and lasting obligations to them for the encouragement I received at their hands. So much for the past and so much for the early history of this operation. To-day it is done in all lands, everywhere. I have reports from far off Brazil of more than one successful case. As much as seven years ago I was startled by receiving a report from an Irish surgeon in our immediate antipodes. Straight down through the globe on the other side stand a few little islands of the sea. An object of special interest were they to me when I was a little school-boy, simply from the habit of its people of regaling their guests, upon festive occasions, upon the delicacy of roasted missionaries; the Fejee Islands, I mean. The idea at that time, seven years ago, of Battey's operation, originating in the little town of Rome, in Georgia, passing around one-half of the entire circumference of the globe and being put in execution upon an invalid Fejee in that far off land, startled me.

The first published case of this operation in Great Britain was by Prof. A. R. Simpson, of Edinburgh, upon a young lady living at Dundee, in Scotland. Simpson gave me the history of the case. He had his patient write me a congratulatory letter, enclosing her photograph, and upon a subsequent visit to Europe he telegraphed her down from Dundee, introducing me to her, giving me an opportunity of verifying fully and completely the case. It was a successful and triumphant cure. Sir Spencer Wells, known to the world for his great and wonderful career in ovariotomy, has been extremely conservative in touching this operation, conservative as he is in everything, but he has found a few cases himself where it was absolutely indispensable for the cure of the patient. He sent me an account of his first case, done at Cannes, in Southern France, on the Mediterranean, and likewise had his patient to write me a congratulatory letter, giving an account of her previous sufferings and her restoration to health. Whilst in Europe, on my last visit in 1881, at the Congress, Sir Spencer Wells did me the honor to call upon me to confer in the case of a titled lady, a viscountess of Great Britain, and verify his diagnosis and recommendation of Battey's operation for her cure. Dr. Matthew Duncan, of London, an old acquaintance and friend whom I very much esteem, for a long time placed himself firmly against the operation and denounced it boldly and unhesitatingly. I had the pleasure of dining with Dr. Duncan, at my visit there at the Congress, and was gratified to learn from his own lips that even he couldn't get along without Battey's operation, for he confessed to me that he had two patients that were submitted to it, not at his hands, but with his approval and sanction, as indispensable for their cure. In Great Britain the operation has been very much done within the last ten years, I might say within the last eight years, by Mr. Tait, Dr. Savage and Dr. Mallins, the three principal operators. The city of Birmingham has taken the lead in Great Britain. London men speak rather flippantly now about the Birmingham School of Gynecology, so-called, and especially of the terrible slaughter of ovaries that they say occurs over there. In London, however, the operation has been done in a number of cases and is a stândard operation now with Thornton and Bantoch, of the Samaritan Hospital for Women, and also by Dr. Haywood Smith and his colleagues in the Hospital for Women at Soho Square.

In America this operation has been quite extensively practiced in New York by Prof. Thomas and Dr. Gill Wylie, who are the principal operators. There are various others there, but the operations are chiefly in the hands of Thomas and Gill Wylie. Dr. Emmett, like my friend, Dr. Matthew Duncan, of London, thoroughly honest men and eminently conservative men on all questions of surgery and medicine, so very erect indeed are both of them that they actually, like the aboriginal Indian, have schooled themselves to lean over backwards. Even Dr. Emmett has found it necessary to do this operation for the relief of a few of his cases, and takes the position that the operation must be accepted, but that it is only to be accepted as a rare necessity. In Philadelphia, Dr. Goodell has done and is doing the operation frequently and has operated quite a large number of cases; I can't say how many. Dr. Montgomery, of Philadelphia, and Meigs Wilson also are doing it, as well as several others. In Baltimore, Dr. Henry P. C. Wilson and Dr. Howard, leading gynecologists of Baltimore, have both frequently done the operation. In the West it has been done a few times, in St. Louis by Dr. Engelmann, but, I am sorry to say, with very unfortunate results. His mortality has been quite large. I have operated myself twice in Minnesota, and I think other surgeons—in fact, I know other surgeons have adopted the operation and are practicing it there as well as in the State of Michigan.

Next, gentlemen, a few words as to the proper field for this operation. In the first place, let me say that the ovaries which are removed in these cases are almost invariably found to be diseased. This disease cannot always be recognized in advance of the operation. Frequently it is impossible to recognize the actual existing condition of the ovaries. Many writers at the present day, and I think Sir Spencer Wells may be ranked among them, and Hegar, of Germany, with very many others, take the ground that the operation is never justifiable unless absolute disease of the ovaries can be diagnosticated beforehand. I take out the ovaries, gentlemen, in many cases where the condition of the organs cannot possibly be known beforehand. The ovaries are buried in inextensive deposits of lymph, covered up completely, so that it is even difficult to recognize them by the sense of touch after the abdomen has been opened. To give you an idea of this difficulty: In one of my cases at Louisville, Ky., a notable case, around which were assembled a galaxy of surgical talent rarely equalled at any operating table in America. In a chair by my side sat the venerable Dr. Gross. My patient was anesthetized with the sponge in the hands of Marion Sims. On my right sat Prof. Lewis A. Sayre, of New York, and assisting me in handling my instruments was Prof. Yandell, of Louisville. When the abdomen was opened (the vaginal opening through the cul de sac), I passed in my finger and explored a great mass of lymph in the pelvis, and with my inexperience at that time I felt myself somewhat in doubt as to the location of the ovaries. I asked Dr. Sims to examine and see if he could locate them satisfactorily. He did so and reported that he could not. He said to me, "Doctor, scratch about a little and you will find them somewhere." I began to scratch about and, sure enough, after a little I brought out on my finger-nail what everybody pronounced to be unquestionably ovarian stroma. That gave me the key and I went on and gouged them out. If Marion Sims was at fault in recognizing the ovaries in such a case, I am sure the gentlemen will excuse me, with my experience at that time, for not recognizing them, and I am sure that the majority of those present would find themselves equally at a loss to recognize the ovaries in many of these cases after the abdomen is opened. If so, how shall we expect to diagnosticate the absolute condition of the ovaries beforehand with the abdominal wall or the vaginal tissues between your index finger and the organs for which you are searching. The idea is preposterous. It will not do, gentlemen, to lay down any such test. The test which I marked out originally, fourteen years ago, for determining these cases I have held to ever since. I hold to it to-day. It is very much better, is less likely to lead you astray than any, so far as I know, that has ever been suggested. That test is this: Your case must answer three questions satisfactorily: First, is this a grave case? If it is not, close up your instrument case and go along about your business. Nobody has any business extirpating a woman's ovaries for trivial causes.

In the second place, is the case curable by the resources of the art within the reach of your patient? If so bundle up your instruments and go your way. Nobody has any business extirpating a woman's ovaries when she can be cured by other means. These propositions seem to me to be self-evident. I think they will rarely lead you astray. Now ask yourself the third question: Is it reasonable, is it physiological, is it logical to expect that the extirpation of the ovaries would probably cure this case? If it is you have got a fair case for operation, whether the ovaries be diseased or not. If it is a grave case of disease and the patient has been years bed-ridden, and has worn out the whole profession of her neighborhood and everybody within her reach, shall she lie the balance of her days hopeless in her bed because Dr. Jones don't chance, on his digital examination, to find her ovaries diseased? I wot not, gentlemen, not at all. If I know that her case is an extreme one, if I know that she is an object of great sympathy and pity, if I know that she is absolutely incurable by the resources of the art, if I have good reason to believe that the extirpation of her ovaries will again restore her to health, to the world and her family, I shall interfere without hesitation. I ask nobody whether her ovaries are diseased or not. Of course, when I take them out, if I find those ovaries diseased, I felicitate myself that I have come within everybody's rule when that is the case, but I should not hesitate a moment, if I was satisfied that the patient was incurable, that her case was an extreme one, that we had hopeful ground to believe that she would be cured by the loss of her ovaries. Each case, then, gentlemen, must stand upon its own merits. This point I made before the last meeting of the American Gynecological Society and was abundantly sustained by the Society at that meeting, and my friend, Prof. Barker, of New York, who is a generous friend, as I have always found him, questioned me specially upon this point.

I see many ladies, gentlemen, in Georgia, suffering with these maladies, bed-ridden, hopelessly so. If it were in my power to transfer them to New York and put them under the tender care of my friend, Dr. Barker, I would not for a moment think of extirpating their ovaries. If I had a sufficient length of purse and could assemble those patients in my infirmary at Rome and subject them to proper uterine treatment, and proper constitutional treatment, and keep them there for one, two and three years, I should certainly do it in many of those cases in place of extirpating their ovaries. But why talk to a patient in the very depths of poverty, unable to afford decent nourishing food for her sustenance, unable to afford a competent nurse, and who has worn out the medical profession in her vicinity until no doctor could be got to visit her, everybody saying it is perfectly useless, I can't waste my time with such a case as that. What are you to do, gentlemen? Send her to New York and put her in the Woman's Hospital, send her to private infirmaries and hospitals of New York? Shall I take her to Rome and keep her at my private cost for one, two and three years? Gentlemen, we just can't do it. There is no use to talk about it. That case is absolutely incurable by the existing state of the art and the means within her reach. Her poverty makes it so. If I step in with my knife, and at the sacrifice of her ovaries restore her to health and to happiness, and make her in the place of an invalid and a tax upon the energies of her poor mother, and brothers, and sisters, watching her by night, agonized by her sufferings, rent in their feelings by her continual torture, and restore her to health and happiness, and make her useful in the household in helping to maintain that family, in the place of being a helpless invalid, I think I have done her a great kindness, even though I do it at the sacrifice of her ovaries. What are her ovaries worth to her, gentlemen, under such circumstances? What boots it to her that my friend, Dr. Barker, of New York, is an eminent gynecologist and obstetrician, that he is learned in all these diseases and has healing balm in his hands to administer to suffering humanity? What boots all that to her? It is entirely irrelevant to the case. That view of the case, gentlemen, was accepted by the most conservative members of the American Gynecological Society. Even my friend, Dr. Emmett, of New York, was obliged to admit it. Let each case present itself on its own merits.

Now, let us touch upon the extirpation of the tubes. My rule is now, as it has always been, to extirpate the fallopian tubes when they are diseased. I have doubts, gentlemen, of the necessity of this. I think it right as a matter of conservatism to do it, on the ground that having removed the ovaries, the tubes are, as the lawyers say, surplusage; there is no special necessity for their longer remaining in the body. If one is diseased it seems well enough to get it out of the way. As a matter of fact I have grave doubts upon the question of necessity, because I believe, after having removed the ovaries and having accomplished the change of life, that the diseases of the tubes, for the most part, would subside of themselves if they were left alone. That is my confident belief; still my practice has been, when the tubes were found diseased, one or both of them, to remove them. Enough of that.

A word now with reference to what I shall style, coining a new term perhaps, the *tubular theory* of menstruation. A few years ago an eminent surgeon put before the profession the ingenious theory that the ovaries were organs of comparative little importance in the female economy, that the great central organ which determined the physiological process of menstruation was not the ovary, but the fallopain tube. He boldly claimed that if the fallopain tubes were removed the presence of the ovaries was a matter of indifference; that the change of life would attend upon the removal of the tubes simply, and that the curative results of the operation would be fully secured, leaving the ovaries behind. Unfortunately, gentlemen, for this theory, as well as many others of the theories of medicine, simple and beautiful as it is, it does not stand the test of experience. It is not supported by the facts, and a theory which is not supported by the facts, especially a theory which is diametrically opposed to the facts, sooner or later falls to the ground. I think I may safely say that the tubular theory of menstruation has fairly fallen to the ground. If anybody defends it now, I do not know the fact. It is a very curious idea to my mind that such a theory should ever have been originated. It seemed to me quite as reasonable to say that the testicle was an organ of very minor importance in man, that the great central organ upon which rested his generative capacity and its attending functions was the *vas deferens* and not the testicle. It seemed to me then quite as reasonable as the tubular theory of menstruation and it seems to me so to-day. But I have wasted words enough on that subject, as nobody, so far as I know, maintains that theory to-day.

A few words now, gentlemen, with reference to antisepsis. This is a hackneyed subject. I do not propose to go into its general consideration. I only propose to call your attention to a notorious fact that coincidently with the application of the antiseptic principle and the antiseptic methods of Mr. Lister, to the operation of ovariotomy came a very great and striking diminution in the mortality of the operation, a wonderful diminution. The mortality of ovariotomy in the hands of Dr. Thomas Keith, of Edinburgh, a very honest, conscientious and most painstaking surgeon, dropped from the extraordinary success he had previously reached with a mortality of ten in a hundred only, when Sir Spencer Wells was reporting a mortality of twenty-five, thirty and thirty-three in a hundred. I say, on the introduction of antisepsis in the hands of Dr. Thomas Keith, his mortality dropped from ten in a hundred down to only two or three in the hundred, a very great fall. Like results have been obtained in the hands of others. Sir Spencer Wells himself was forced to take it up and a like improvement in his results occurred, although never quite equal to those of Dr. Keith. Such has been the history all the world over. Up to the introduction of antisepsis in ovariotomy, the operation was little short of murder, in Germany little short of murder for a long time. The deaths from ovariotomy in portions of Germany were from sixty to seventy, I believe, in the hundred. In Italy it was even worse than that. For a long time so many women as had their bellies ripped open in Italy, so many women went straight to the church-yard. The mortality was fearful. They saved nobody. After the introduction of antisepsis the operation became a recognized one and is so to-day in Italy. It is very extensively done in Germany and with very excellent results. Not quite equal to Great Britain, but I think I do not overstate the case when I say that they are quite equal to and perhaps a little better than the average in America.

Gentlemen, the scientific questions involved in bacteria, in all the germ theory of disease, in the power of certain chemicals to destroy these germs and prevent their ravages, and all that sort of thing, I leave with the physiologists. I am not a scientific physiologist myself, and I am too old now to become such. All these questions present themselves simply as practical questions with me. My mission for the balance of my life is to serve my race and try to assuage human suffering and save human life if I can. It is a practical view I take of all this subject, and therefore I have not a word to say to the theory of antisepsis. I can only say this, that the facts show that the use of carbolic spray of carbolic solutions, the use of the spray in any form or of any sort of antiseptic solutions, has been shown not to be indispensable to successful ovariotomy. That fact I think is indisputable, but as a practical man, when I see my own results dropping from a mortality of 20 to 25 per cent. down to a mortality of 3 or 4 per cent., the methods that have been employed coincidently with that remarkable improvement in my success commend themselves to my judgment. I feel very loth indeed to change either the dotting of an i or the crossing of a t in the programme as long as I get such results. Let other gentlemen discuss the scientific questions at their leisure. I have not time for that. I therefore use the spray, the carbolic solutions and the carbolized ligatures, because they seem connected with my improved results. I have no war with any gentleman on the scientific questions that may be involved in the subject.

One word, gentlemen, as to the conditions of success, as I deem them, and that is the necessity of giving special attention to the minute details connected with these operations, and carrying out all the conditions of cleanliness, of order and of method in their minutest details, so as to insure the successful results, and the success depends upon the careful observance of those details. When Mrs. Battey, whilst an operation is going on, drops a ligature silk upon the floor and picks it up, I shake my head at her, and tell her to put that in the fire. If she says "this is good silk," I reply, put that in the fire. "Its no use to waste that silk." Put it in the fire. There is no compromise in that—put it in the fire. If any of my attendants touch anything that is at all to be apprehended as a source of sepsis, whatever may be the emergency, that individual does not touch about the wound of my patient anywhere or any of my apparatus, bandages, ligatures and instruments until he has undergone a thorough purification. If I were left by myself he couldn't touch a thing. There are many of these details which it is utterly and entirely impracticable to carry out at the residence of your patient. I defy any man to get a successful record in ovariotomy who visits about, like Washington Atlee did, on the cars and from house to house and operate upon the patient in her own bed and in her own house. No man can get statistics in that way. Another thing, gentlemen: In my early practice I was not only in the habit of visiting patients and operating upon them at their own residences and in their own beds, but, looking to the family to provide food and nursing and whatever was necessary for their well-being. I feel very differently about that now. When I put my knife into a patient I am in for the war. Whatever is necessary to make her thoroughly comfortable, whatever is necessary to give her a thoroughly clean bed and clean clothing and good nourishing food, the best that is to be had, and for an indefinite length of time, it comes from sombody's pocket, from mine if there is nobody else. I cannot afford to allow my patients to be neglected. I cannot afford to allow my patients to suffer. In other words, gentlemen, the conditions that are necessary for a high degree of success in this operation are only to be had in establishments set apart for that purpose. We are all familiar with the amount of skill one acquires who gives exclusive attention to diseases of the eye. We are all aware how much superior are the statistics of ophthalmic surgeons who do a large work in cataract operating. Every ordinary family physician has now and then a case of cataract, and in my retired locality I feel the necessity. It is a charity, indeed, sometimes for me to remove a cataract, but never from a patient who has any means to pay even railroad fare down to Atlanta, never, never. I never advise any patient to put himself in my hands for a cataract extraction. My conscience will not allow that. When he or she are within reach of Atlanta, where you have specialists who make that their business, and they are skilled and successful in doing this operation, why should I undertake to operate at Rome? It would be very bad policy; it is very bad humanity; it is very bad morality; at least I think so.

Some months ago a professional friend of mine sent me a young girl with a large ovarian tumor. I turned the tumor out for her and sent her home in about three weeks sound and well. It had been his first case of ovarian tumor. In three or four months he sent me another, and I removed her tumor and sent her home so quick it made his head swim, and he said to himself, "Why, I am losing lots of thunder; why shouldn't I be in this business?" Before the year was out he had another case, and wrote me his thoughts and wanted some suggestions as to the operation and the list of instruments he should buy from New York. He wrote to me that his patient was very poor, that her friends were barely able to pay her traveling expenses to Rome, and would certainly not be able to pay anything for her board or medical attention, and he felt, on the whole, it was rather his duty to operate on her. I replied to his letter and gave him very frank, and what I thought to be excellent, advice. I said to him, Doctor, suppose she gets well, the chances are in all probability you will never get another case in your retired country locality. You have had three already, you have exhausted that crop, there won't be any more there in all probability in your lifetime. It will be ten or twenty years at least before you get another case. You have nothing to gain really by doing the operation. Suppose your patient dies, you will have very much to lose. Your neighbors will say that you "sent two cases down to Rome and they hardly missed them before they were back well, and the third case came into your hands and you operated on it and she is dead. We told you so. We knew it would be that way." It will hurt you. You had better let it alone. Well, he said: "Dr. Battey is ambitious to be a specialist and he wants to gobble up all these cases and get them all down to his infirmary; I don't see why I shouldn't do my share." He called his brethren around him and put the patient upon the table, and unfortunately got into one of those nasty cases, as Dr. Keith calls them in his expressive Scotch phraseology with universal adhesions everywhere, and he began to tear and tear, and the blood began to well up profusely from the abdomen, and, to make a long story short, his patient died on the table, or quickly after they got her in bed, and the result was there was much talk and a stink about it. It got into the newspapers and it hurt him badly. It will take him years to get over it. There is no profit in this sort of thing. This is not my habit in ophthalmic surgery. It must be a clear case of necessity, and of charity too, that puts my knife into a cataract. I have done it repeatedly, and have some successful cases, but I don't do it if I can help it.

A word with reference to the abuses of the operation. This is a much more live question at the North than it is with us. It is going to be quite a live question here also in time. These Northern ladies don't like large gangs of little brats tottling about at their heels; they don't like it at all, and some of them will resort to all sorts of expedients to get rid of a nuisance of that sort, and they will prevent it if they can. Under the pupilage of their sisters from abroad, they have devised, as you all know by hearsay, various ways and means and schemes to accomplish this purpose, and they are already beginning to seek shelter under my skirts to get out of such troubles. This is not a live question with us here at the South, but it will come after awhile. We cannot watch too carefully at that point. More than that, gentlemen, there is an abuse of the operation that ought to be guarded in another direction. A physician gets tired of his patient; he has exhausted his skill upon her and he flatters himself that his skill is as good as anybody's skill, and whether it is or not, it is not for me to gainsay it. But there is luck in some men. I have cases sent to me that have exhausted the skill of wiser and better men than I am; sent for the extirpation of their ovaries, and I look them over and take a different view of the case from that of the attending physician, and think I can see a way out of it, and I put the patient in my infirmary and treat her after my usual methods there, and by and by she gets into a very comfortable and good state of health, and I send her home and the doctor is very much surprisd to find her better. I see such cases every now and then. So if a patient has been two or three years in bad health and has not recovered and is in a very bad way, that does not justify the extirpation of her ovaries. There ought to be something more than that. It ought to be clear that she has had a fair chance to get well without it. Ought to be clear, as I said before, that the case is an extreme one. There is such a mania for a knife, itching fingers, as it were. There is a certain class of men who *seem* to love to cut people. They don't really love to cut people, but they have a yearning desire, as they say, for something that will bring them prominently before the community; and when they cut people in a bold and daring sort of way it frequently does get them into the newspapers and bring them prominently before the community. It is needless to say that the true surgeon harbors no such instinctive longing for notoriety as that, and learns to forget himself and the possible effect of a little notoriety upon his professional business, absorbed all the time in a self-sacrificing desire to faithfully discharge his duty towards his patient. There, I conceive, is the ground-work of all true success in medicine, of all true success in surgery.

To the younger gentlemen present, if you will allow me to drop a word in your ears, if you want to succeed in life, in professional life I mean, don't be too careful when a call comes to you to inquire into the circumstances of your patient, whether he is able to pay a good fee or not. Don't be too careful to prune closely at the outset and trim your practice into influential patients only, and all that sort of thing. Try to infuse within your own heart and soul a true spirit of benevolence, love of your kind, zeal in your profession, anxiety to relieve human suffering, and if you pursue your mission with your whole heart, with true earnestness of purpose, *somebody* will find it out, and it will not be a great while before a great many people will find it out, and they are not going to let you starve. That sort of men is too scarce to let starve. They don't starve in America. They can't be spared. If you want to be sure of your bread and meat and provender for your horse and something for the blacksmith and carriage man, take that recipe and try it awhile. I think I can say confidently, gentlemen, from the very first day that I practiced medicine it has been a rule with me to give no thought for the morrow, what I should eat, wherewith I should be clothed. Consult the interests of your patients. Try and get them well in the shortest possible time and somebody will clothe and feed you and you will have an established practice and an established reputation. You will have the support and confidence of the community in which you live.

A few words with reference to the horoscope of the future. In spite of all we can do, this operation, like almost any operation known to surgery, is obliged to be overdone. It is so with all new remedies in medicine. They take a run, they are fashionable. This operation is a ready resource; it is a relief from dilemma and we must expect nothing else but that it will be overdone. We ought to guard this point as carefully as we can, but the consumers of the medical world will enter into judgment on these things and eventually the operation will settle down into its proper place where it belongs. Whenever that day arrives I believe unequivocally that it will be permanently fixed for all time amongst the resources of surgery. I do not think that medicine will ever reach that point of perfection when we can dispense with the knife in very many of these cases. Of course, as argued by Dr. Emmett, Battey's operation is a humiliating confession of weakness to the medical profession. That is self-evident. If medicine was perfect there would be no use for Battey's operation. Nothing that is human can ever reach that point of perfection. Science itself has its bounds and limits and it must always be so, and, therefore, the time will never come when there will not be a place somewhere for Battey's operation.

